# Diabetes mellitus promotes the nasal colonization of high virulent *Staphylococcus aureus* through the regulation of SaeRS two-component system

**DOI:** 10.1080/22221751.2023.2276335

**Published:** 2023-11-08

**Authors:** Qichen Wang, Nadira Nurxat, Lei Zhang, Yao Liu, Yanan Wang, Lei Zhang, Na Zhao, Yingxin Dai, Ying Jian, Lei He, Hua Wang, Taeok Bae, Min Li, Qian Liu

**Affiliations:** a Department of Laboratory Medicine, Ren Ji Hospital, School of Medicine, Shanghai Jiao Tong University, Shanghai, People’s Republic of China; b Department of Vascular Surgery, Yueyang Hospital of Integrated Traditional Chinese and Western Medicine, Shanghai University of Traditional Chinese Medicine, Shanghai, People’s Republic of China; c Department of Otorhinolaryngology, Head and Neck Surgery, The Second Hospital of Anhui Medical University, Hefei, People’s Republic of China; d Department of Microbiology and Immunology, Indiana University School of Medicine-Northwest, Gary, IN, USA; e Faculty of Medical Laboratory Science, Shanghai Jiao Tong University School of Medicine, Shanghai, People’s Republic of China

**Keywords:** Diabetes, *Staphylococcus aureus*, colonization, SaeRS two-component system, microbiome

## Abstract

Diabetic foot infections are a common complication of diabetes. *Staphylococcus aureus* is frequently isolated from diabetic foot infections and commonly colonizes human nares. According to the study, the nasal microbiome analysis revealed that diabetic patients had a significantly altered nasal microbial composition and diversity. Typically, the fasting blood glucose (FBG) level had an impact on the abundance and sequence type (ST) of *S. aureus* in diabetic patients. We observed that highly virulent *S. aureus* ST7 strains were more frequently colonized in diabetic patients, especially those with poorly controlled FBG, while ST59 was dominant in healthy individuals. *S. aureus* ST7 strains were more resistant to human antimicrobial peptides and formed stronger biofilms than ST59 strains. Critically, *S. aureus* ST7 strains displayed higher virulence compared to ST59 strains *in vivo*. The dominance of *S. aureus* ST7 strains in hyperglycemic environment is due to the higher activity of the SaeRS two-component system (TCS). *S. aureus* ST7 strains outcompeted ST59 both *in vitro*, and in nasal colonization model in diabetic mice, which was abolished by the deletion of the SaeRS TCS. Our data indicated that highly virulent *S. aureus* strains preferentially colonize diabetic patients with poorly controlled FBG through SaeRS TCS. Detection of *S. aureus* colonization and elimination of colonizing *S. aureus* are critical in the care of diabetic patients with high FBG.

## Introduction

Diabetes mellitus (DM) is a complex metabolic disorder characterized by hyperglycemia resulting from decreased glucose metabolism [1]. The worldwide estimated number of diabetes patients is expected to reach 700 million by 2045 [2]. Diabetic foot ulcers (DFU) are a common complication of diabetes, beginning with neuropathy and resulting in foot ulcers [3]. DFU is a costly and prevalent complication in diabetes patients, leading to a higher risk of death within five years [4]. It has been reported that foot ulcers are more prevalent in type 2 diabetes [5], and they are difficult to treat due to impaired healing processes and decreased immune responses [6]. Furthermore, over half of foot ulcers become infected, often leading to lower extremity amputation [7]. Therefore, controlling blood glucose levels, patient education, footwear interventions, and understanding wound site infections are crucial for effective prevention of foot ulcers [8].

Diabetic foot infections (DFI) are mainly polymicrobial, and *Staphylococcus aureus* is the most frequently isolated pathogen in diabetes-related infections [9–11]. *S. aureus* is a nostrious pathogen which can cause many diseases including skin and soft tissue infection, pneumonia, sepsis, etc., [12]. The success of *S. aureus* as a pathogenic bacterium is due to the production of numerous virulence factors, which are controlled by multiple regulators, including the Agr quorum sensing system, the SaeRS two-component system (TCS), and the toxin repressor Rot [13]. The sensor kinase SaeS autophosphorylates its hisdine residue, and transfers the phosphoryl group to the response regulator SaeR. The phosphorylated SaeR modulates the transcription of the virulence genes by binding to the corresponding promoters [14]. According to the different affinity of phosphorylated SaeR and target genes, two classes of SaeRS target genes are classified: the low affinity targets (i.e. *splB*, *hlgA*, *emp*, *eap*, and *fnbA*), which can be activated by the induction of SaeRS TCS; and the high affinity targets (i.e. *hla*), which only require the basal level of SaeR phosphorylation [15]. Previously we reported that SaeRS TCS contributes to the age-dependent nasal colonization of *S. aureus* [16]. The epidemiological analysis showed that the clinical phenotypes, severity, and outcome of diseases caused by *S. aureus* are affected by the bacterial clone types [17]. The variation of *S. aureus* strains affects the outcome of diabetic wounds [18]. However, the origin of these strains is still poorly understood.

*S. aureus* can colonize in human anterior nares asymptomatically. The nasal *S. aureus* strains causing diabetic foot ulcers have been reported [12,19,20]. We suspected that the clonal types of *S. aureus* strains in the nasal microbiome might affect the infection severity and prognosis in diabetic patients. However, little is known about the nasal microbiome, especially the colonization of *S. aureus* in diabetic patients. In the study, we investigated the nasal microbiome of type-2 diabetes patients by 16S rRNA sequencing and culture-based analysis. Moreover, the dominant sequence types of *S. aureus* for their colonization were characterized by competition assay both *in vitro* and *in vivo*. Finally, the pathogenic traits of *S. aureus* were detected using animal infection model.

## Materials and methods

### Ethics statement

The animal experiment was performed following the Guide for the Care and Use of Laboratory Animals (Eighth Edition). The human and animal study were approved by the ethics committee of Renji Hospital, School of Medicine, Shanghai Jiao Tong University, Shanghai, China. All individuals provided informed consents.

### Participant enrolment and nasal swab collection

In total, 149 type-2 diabetes patients (DM) and 174 non-diabetes (NDM) volunteers from the community of Shanghai, China, were enrolled for the research. The exclusion criteria for the study were as follows: use of immunosuppressive agents normally, history of rhinitis or other chronic nostril diseases, carriage of medical devices, use of any antimicrobials within one month preceding enrolment. Nasal swabs were immediately submerged in 1 ml sterile saline after collection.

### DNA extraction and 16S rRNA gene sequencing

After vortexing for 2 min, the swabs were centrifuged at 13,000 × *g* for 10 min, 4°C, upon which the pellets were dissolved in Buffer ALT (QIAamp DNA Mini Kit, Qiagen 51306) with lysozyme (1.25 mg/ml, Sigma L6876) and lysostaphin (25 μg/ml, Sigma L4402) and incubated for 30 min at 37°C. DNA extraction was performed according to the protocol of the QIAamp DNA Mini Kit. 16S-rRNA gene libraries were generated by PCR from purified genomic DNA with primers 341F (5′-CCTACGGGNBGCASCAG-3′) and 805R (5′-GACTACHVGGGTATCTAATCC-3′), which amplify the hypervariable V3-V4 regions of the 16S rRNA gene. The amplicons were further sequenced on the Illumina Hiseq 2500 platform.

### Sequence analysis

After trimming with the FastX tool kit, the sequencing reads with a Phred base quality above 25 and read length longer than 30 were used for assembly using Flash software [21]. The chimeric sequences was filtrated by USEARCH 8.0 implemented in QIIME [22]. After splicing, the amplified sequences were between 400 and 500 base pairs for all samples. After exclusion of the samples with reads of less than 10,000, 280 samples (130 in the DM group and 150 in the NDM group) were amplified successfully. The Ucluster algorithm implemented in QIIME was used for operational taxonomic units (OTUs) cluster at a similarity level of 99% [23]. The Silva database (https://www.arb-silva.de/documentation/release-128/) was used as a reference for the RDP classifier with a confidence cutoff of 0.8. Beta diversity indices [Principal coordinate analysis (PCoA) and Bray–Curtis dissimilarity] were calculated at a 97%-similarity cutoff by QIIME. The abundance of each OTU was compared using the subset of 10,000 random reads for each sample. The 16S rRNA sequencing data is available in the Sequence Read Archive (the accession number: PRJNA929354).

### Taqman qPCR assay

Concentrations of *S. aureus* DNA were determined using quantitative TaqMan real-time PCR targeting the single-copy gene. The nuclease (*nuc*) gene of *S. aureus* was used for amplification [24]. The qPCR was performed in a 20 μl reaction volume containing 2× KAPA PROBE FAST qPCR Master Mix, primers, TaqMan probe (TGCATCACAAACAGATAACGGCGTAAATAGAAG), and 4 μl of DNA template. The total bacterial loads were determined by testing the expression of 16S rDNA [25]. The negative control contained sterile distilled water instead of template DNA. Amplification was performed on the 7500 Real-Time PCR system. The relative bacterial contents were determined by comparing with the total bacterial loads for every sample. The loads of *S. aureus* were determined using the standard curves, which was evaluated by adding the templates consisting of *S. aureus* cells at known concentrations.

### Bacteria

All the nasal swabs from recruitments were submerged in 1 ml sterile saline. After votex, 100 μl samples were cultured on sheep blood agar at 37°C for 24 h for bacteria isolation. At least 20 colonies were selected and subjected to species identification using MALDI-TOF-MS (Bruker Daltonics, Bremen, Germany). Briefly, after spotting onto the steel target plate, the bacteria were added with 1 μl 10% formic acid (Sigma F0507) and dried for 5 min at 75°C. After adding 1 μl MALDI matrix [a saturated solution of a-cyano-4-hydroxycinnamic acid (Sigma 70990) in 50% acetonitrile / 2.5% trifluoroacetic acid] to the bacteria, the plate was subjected to the MALDI-TOF MS system for analysis. The spectrum was obtained in linear positive-ion mode range from 2000 to 20,000 Da. Each spot was measured manually on five different positions by using 1000 laser shots at 25 Hz in groups of 40 shots. The spectra was analysed by MALDI Bruker Biotyper 3.0 software and library (Bruker Daltonics). Among all the *S. aureus* strains, we randomly selected 60 strains from each group with a random number generator (Rand function of Perl5) (Table S1). For RT–PCR and animal experiments, 9 isolates of ST7 and 9 isolates of ST59 were randomly selected from DM and NDM group respectively.

### Mouse strains, husbandry, and genotyping

The male *db*/*m* (C57BLKS/JGpt) and *db*/*db* (Strain NO. T002407, BKS-Lepr^em2Cd479^/Gpt) mice (eight-week-old) were purchased from GemPharmatech Co., Ltd (Nanjing, China). The mice were housed under specific pathogen-free conditions in filter-top cages that were changed bi-monthly by veterinary or research personnel. Sterile water and food were provided ad libitum. Mouse age, sex, lineage, and source facility were tracked for all experiments.

### Mouse skin abscess model

Bacterial cultures were spun down and washed with sterile phosphate-buffered saline (PBS) before resuspension for infection. The mice were anesthetized with Avertin (Sigma T48402) by intraperitoneal injection; then, the hair was shaved on the back for *db*/*m* and *db*/*db* mice. The mice were administered (10^8^ CFU in 100 μl of PBS) by s.c injection. Each day the skin lesion size was measured by length (L) and width (W). The mice were sacrificed on the 2nd day. the skins were surgically removed by a 8-mm punch and homogenized in 500 μl PBS. The homogenized tissues were diluted and then spread on 5% sheep blood agar for CFU determination. The histological analysis was analysed by hematoxylin & eosin (H&E) staining after paraffin embedding.

### Real-time quantitative reverse transcription-PCR

For bacteria: The *S. aureus* isolates were grown to an exponential phase. For animal tissues: The skin tissues were homogenized and resuspended in RLT buffer from the kit (Qiagen). The cell pellets or tissues were broken with a FastPrep-48 (MP Biomedicals Products) at 800 rpm for 300 s three times. After centrifugation, the supernatant was collected for total RNA isolation according to the manufacturer’s instructions (Qiagen). 1 μg of total RNA was treated with DNase and reverse transcribed with a Prime Script RT reagent kit (Qiagen). The RT–PCR was tested using cDNA as a template with SYBR Green PCR reagents (Roche). Reactions were performed in a MicroAmp optical 96-well reaction plate with a 7500 sequence detector (Applied Biosystems). The primers were listed in Table S2.

### Competition assay *in vitro*

The bacteria pairs used for competition assay were listed in Table S3. Here *S. aureus* ST7-n4343-1 from DM group and ST59-n4225-5 from NDM was used as an example. Bacteria were grown in exponential condition in TSB. After washing in PBS for 3 times, the bacteria was adjusted to the same load (OD_600_ is around 0.5) in synthetic nasal medium (SNM3). *S. aureus* ST7-n4343-1 and ST59-n4225-5 were mixed at 1:1 ratio and incubated in SNM3 with different glucose contents with shaking. After co-culture for 6 h, the bacteria was plated to determine CFU on agar plate with or without erythromycin (10 μg/ml) for strain distinction (ST59-n4225-5 is resistant to erythromycin).

### Competitive nasal colonization model

The bacteria were prepared as above. ST7-n4343-1 and ST59-n4225-5 or the corresponding deletion mutant strains were mixed equally (10^7^ CFU for each). The mice were anesthetized by intraperitoneal injection with Avertin, and 20 μl aliquots containing 10^7^ CFU were instilled intranasally. The animals were sacrificed on the 2nd day after instilment. The noses were surgically removed and homogenized on ice in 0.5 ml of PBS using a manual homogenizer (Tiagen). The homogenized tissues were diluted and plated on TSB agar with or without erythromycin (10 μg/ml) for strain distinction and determination of CFU.

### Statistical analysis

Statistical analysis was performed using GraphPad Prism Version 9.0 or QIIME (Statistical analysis is by 1-way ANOVA and Tukey’s post-test.). All error bars show the mean and standard deviation (SD). The number of replicates or animals used is given in the figure legends.

## Results

### Demographic analysis

We recruited a total of 323 seniors, of which 149 were diabetes patients (DM, 72.88 ± 6.54 years old) and 174 were not (NDM, 70.95 ± 6.76 years old). The seniors commonly suffer from several diseases, such as chronic pulmonary disease, chronic heart disease, chronic renal disease, chronic liver failure, cerebrovascular disease, neoplastic disease, hypertension, neurological disease, and digestive system disease. When the incidence of these diseases was compared between the NDM and the DM groups, cerebrovascular disease and hypertension were significantly more prevalent among the DM patients (Table S4).

### The composition of nasal microbiome in diabetic patients

**Figure 1.**
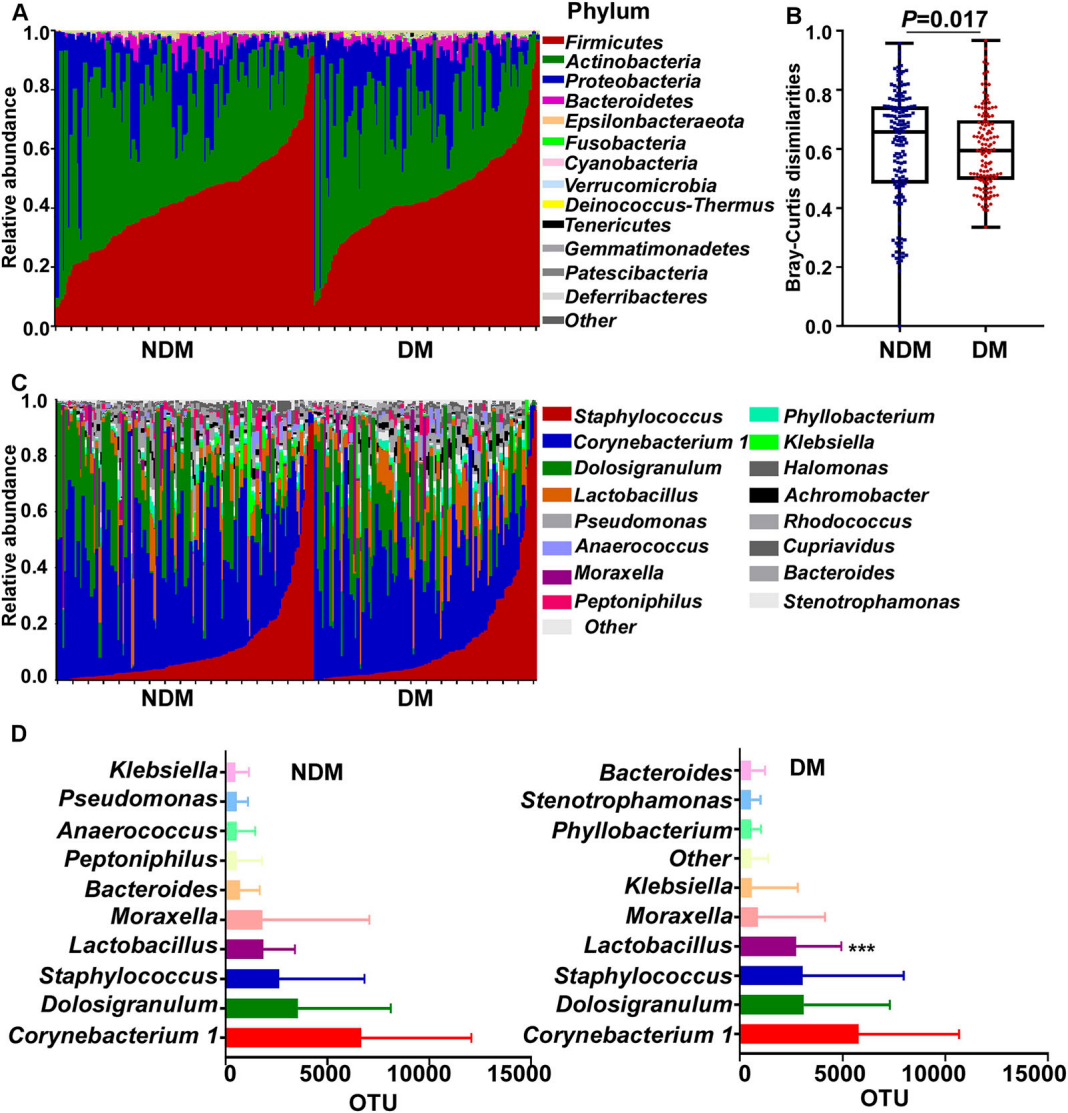
The nasal microbiome composition of the test subjects. (A) Relative abundance of the major phyla in the NDM and the DM groups. (B) Bray-Curtis dissimilarity of the two groups (phylum level). Statistical analysis was done by 1-way ANOVA and Tukey’s post-test. Error bars show minima and maxima. (C) Relative abundance of major genera in the nasal microbiome of single individuals in the NDM and the DM groups. (D) The abundance of major genera in the NDM and the DM groups. Bars depict means; error bars indicate SD. NDM, non-diabetes; DM, diabetes. Statistical evaluation was analysed by ANCOM. ***P < 0.001.

The overall composition of the nasal microbiome was determined by 16S rRNA sequencing and compared between the NDM and the DM groups. Analysis of the major phyla suggested that the microbial composition and diversity were significantly changed between the NDM and the DM group (Figure 1A&B). In general, *Corynebacteria*, *Dolosigranulum*, and *Staphylococcus* were the top 3 genera in both groups (Figure 1C&D). Although *Firmicutes* is the predominant phylum, at the genus level, *Corynebacterium,* which belongs to the phylum *Actinobacteria,* was the most prevalent in both groups (Figure 1D). Among the top 10 genera, *Lactobacillus* was significantly more abundant in the DM group than in the NDM group (Figure 1D).

### S. *Aureus* is more abundant in DM patients

**Figure 2.**
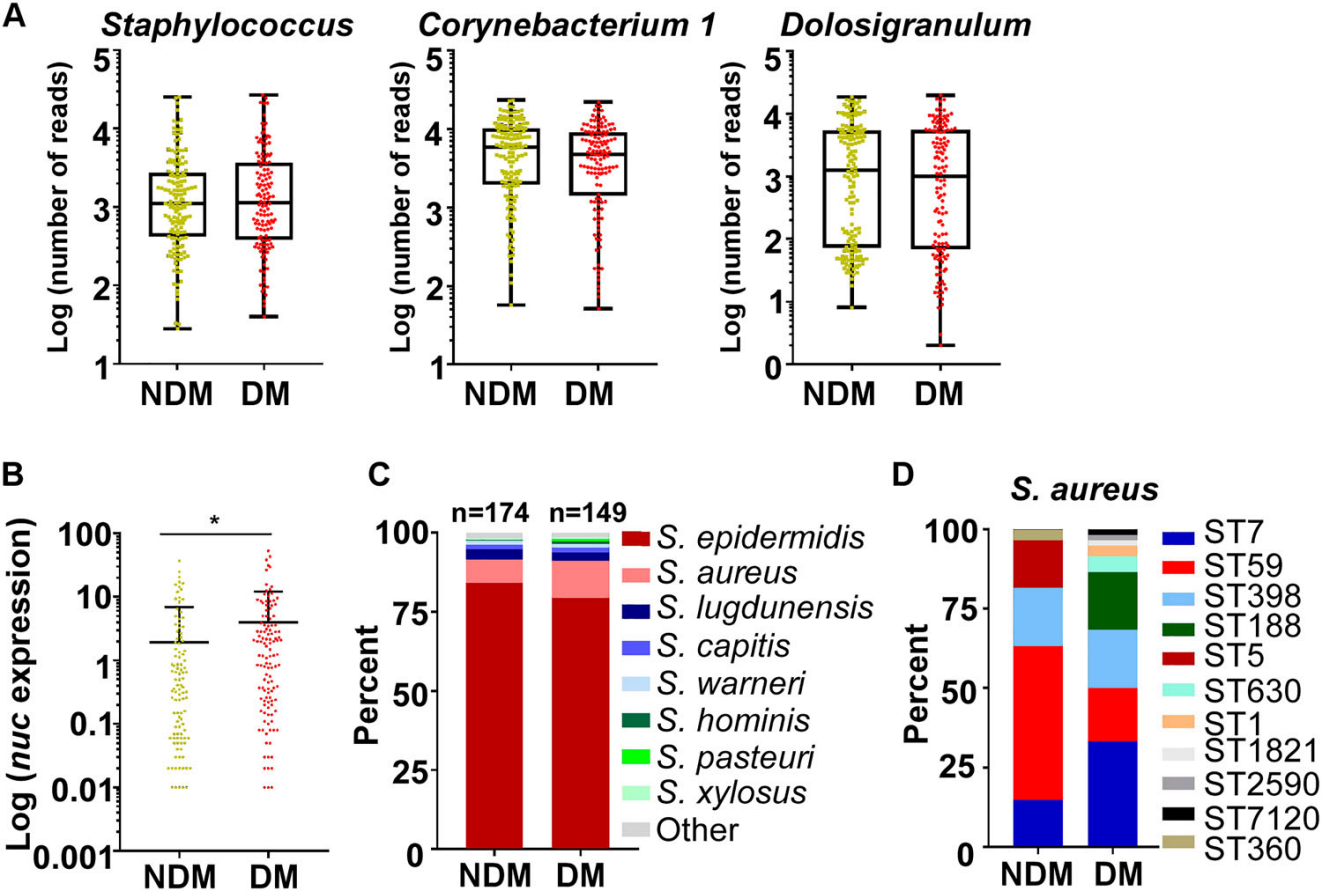
Distinct sequence types of *S. aureus* colonize the NDM and the DM groups. (A) The abundance comparison of the top three genera. (B) The abundance of *S. aureus* was assessed by TaqMan qPCR assay of the nuclease (*nuc*) gene. The results are presented as the mean ± SD. Statistical significance was evaluated by the Kruskal-Wallis test, followed by Dunn’s multiple comparisons test. *P < 0.05. (C) The culture-based analysis of *Staphylococcus* species in the NDM and the DM groups. (D) The MLST types of 60 *S. aureus* strains selected from each test group. Sixty strains were randomly selected and tested by PCR. NDM, non-diabetes; DM, diabetes.

The abundance of the predominant genera was not significantly different between the NDM and the DM groups (Figure 2A). Since *S. aureus* infections are frequent in DM patients, we determined the abundance of *S. aureus* by TaqMan qPCR targeting the nuclease (*nuc*) gene expression. Intriguingly, *S. aureus* appears to be significantly more abundant in DM patients (Figure 2B).

The higher abundance of *S. aureus* in the TaqMan analysis implies that the composition of staphylococcal species might be different in the NDM and DM groups. To test this conjecture, the relative abundance of staphylococcal species from the original samples was analysed by culture. Coinciding with the TaqMan analysis, the abundance of *S. aureus* was significantly higher in the DM group, which was accompanied by a decrease in *S. epidermidis* (Figure 2C). We further determined the sequence types of the *S. aureus* strains. Sixty *S. aureus* isolates were randomly selected (Table S1). While the considerable heterogeneity was observed, the dominant *S. aureus* ST was different between the groups: ST59 was dominant in the NDM group, whereas ST7 was dominant in the DM group (Figure 2D).

### Blood glucose levels can affect the colonization of *Staphylococcus* ST types

**Figure 3.**
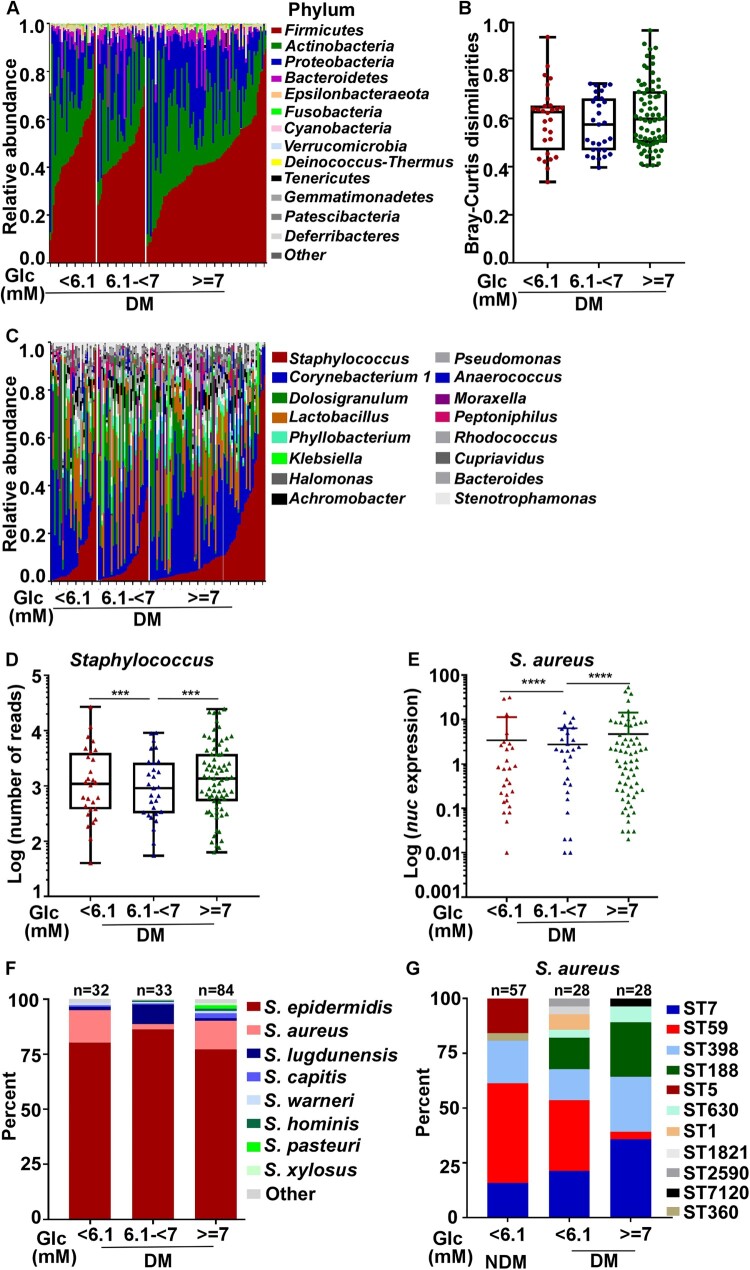
The effect of fasting blood glucose (FBG) concentration on bacterial colonization. (A) Relative abundance of the major phyla in investigated individuals with different glucose concentration in the DM group. (B) Bray-Curtis dissimilarity of individuals with different blood glucose concentration in the DM group (phylum level). Statistical analysis is by 1-wayANOVA and Tukey’s post-test. Error bars show minima and maxima. (C) Relative abundance of major genera in the nasal microbiome of single individuals in the DM group. (D) Abundance of *Staphylococci* of single individuals in the DM group. Error bars show minima and maxima. Statistical evaluation was analysed by ANCOM. ***P < 0.001. (E) The abundance of *S. aureus* was determined in diabetes patients with different FBG concentration using a TaqMan qPCR assay targeting the nuclease (*nuc*) gene. The results are presented as the mean ± SD. Statistical evaluationswere performed using the Kruskal-Wallis test following by Dunn’s multiple comparisons test. ****P < 0.0001. (F) The culture-based analysis of *Staphylococcus* species in diabetes patients with different FBG concentration. (G) *S. aureus* MLST typing was analysed in the NDM group with normal FBG level and diabetic patients with different FBG concentration . Glc, glucose.

The higher nasal colonization of *S. aureus* in the DM group (Figure 2B) suggests that the nasal colonization of *Staphylococcus* might be affected by blood glucose level. In our study, most diabetic patients controlled their fasting blood glucose levels (FBGs) within the normal range (FBG < 6.1 mM); however, some patients didn’t, and their FBG remained high (FBG ≥ 7.0 mM). Therefore, we divided the diabetic patients into three groups by their FBG levels: normal, FBG < 6.1 mM; impaired glucose tolerance (IGT), 6.1 mM ≤ FBG < 7.0 mM; poorly controlled, FBG ≥ 7.0 mM. On a phylum level, the overall composition of the nasal microbiome was not significantly different among the groups (Figure 3A&B). Interestingly, the abundance of *Staphylococcus* genus was substantially lower in the IGT group (Figure 3C&D). TaqMan qPCR assay of the *nuc* gene also showed that the abundance of *S. aureus* was the lowest in the IGT group (Figure 3E). On the species level, the IGT group showed the highest *S. epidermidis* and the lowest *S. aureus* levels (Figure 3F). Although intriguing, we did not further investigate the lower *S. aureus* colonization in the IGT group because of the small sample size (7 *S. aureus* isolates; 4 ST7 in the DM group and 3 ST59 in the NDM group). To our surprise, when the STs were determined for *S. aureus* strains in the normal and the poorly controlled FBG groups, the normal FBG group showed ST composition similar to that of the NDM group in terms of the ratio of ST7 and ST59 (Figure 3G). ST59 was dominant in both NDM and the normal FBG group, whereas, in the uncontrolled FBG group, ST7 was dominant. These results indicate that FBG levels could affect the colonization of *S. aureus* ST strains.

### The antimicrobial activity of coagulase-negative *Staphylococcus* alone cannot explain the suppressive effect on the colonization of *S. aureus*

**Figure 4:**
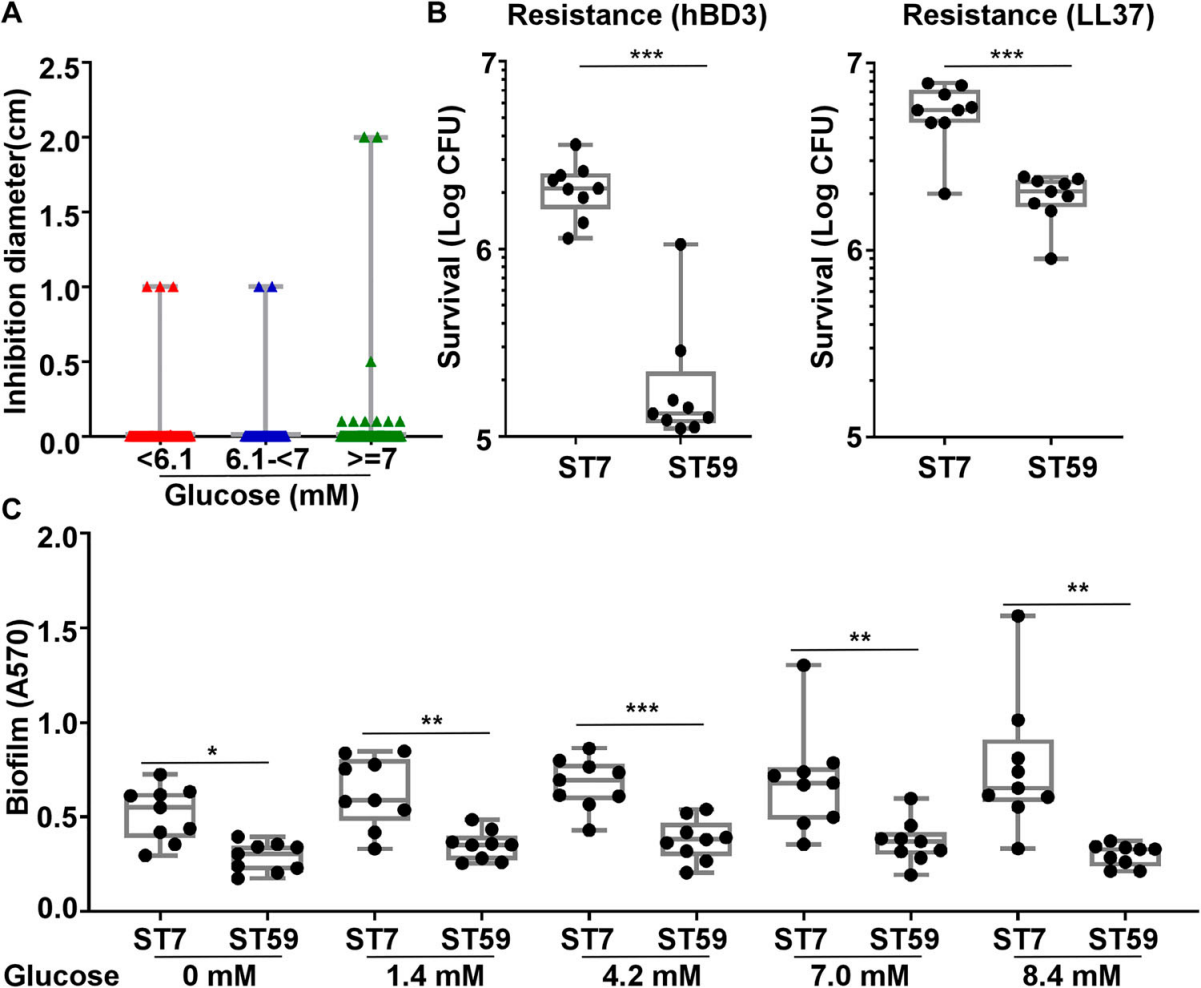
*.*#The antimicrobial resistance and biofilm formation of *S. aureus* ST7 and ST59 strains. (A) The antimicrobial activity of coagulase-negative *Staphylococcus* is not significantly different among isolates from patients with different levels of fasting blood glucose levels. (B) The resistance to two human antimicrobial peptides was compared for 9 randomly selected ST7 and ST59 strains. The bacteria were grown to the exponential growth phase and mixed together with hBD3 (75 μg/ml) or LL37 (75 μg/ml). After 3 h incubation at 37°C, bacteria were spread on sheep blood agar for CFU counting. (C) The randomly selected strains were also subjected to the biofilm formation assay. The strains were grown in TSB with different concentrations of glucose at 37°C for 24 h in a 96-well plate. The data were collected from three biological repeats. The statistical significance was measured by unpaired, two-tailed Student’s *t* test. *P < 0.05; **P < 0.01; ***P < 0.001.

In the culture-based analysis, the abundance of *S. aureus* seems to show an inverse relationship to that of coagulase-negative Staphylococci (CoNS) (Figure 2B). Since CoNS produce bacteriocins, which can inhibit *S. aureus* growth [26], we hypothesized that the bacteriocin produced by CoNS limits the colonization of *S. aureus*. To test this hypothesis, we tested 240 *S. epidermidis* isolates and all 78 other CoNS isolates for their antimicrobial activities by measuring the growth inhibition zone of *S. aureus in vitro*. To our surprise, only 8 isolates inhibited the growth of *S. aureus* (Figure 4A). Among the 8 CoNS with antimicrobial activity, 7 *S. epidermidis* (3 from the normal FBG group, 2 from the IGT group, 2 from the high FBG group) displayed antimicrobial activity against *S. aureus*. The MLST of the 7 *S. epidermidis* strains with antimicrobial activity was diverse, including ST820, ST16, ST210, ST248, and ST20. There was no significant difference in the bacteriocin production among the three FBG groups, suggesting that the bacteriocin production alone cannot explain the apparent suppressive role of CoNS on the colonization of *S. aureus*.

### *S. aureus* ST7 strains are more resistant to human antimicrobial peptides and form biofilm better than ST59 strains

The antimicrobial peptides (AMPs) produced by epithelial cells are important innate immune defenses against pathogens. To examine whether resistance to AMPs is related to the nasal colonization of specific STs, the randomly selected ST7 and ST59 were compared for their resistance to two major human AMPs: hBD3 and LL37. As shown, most ST7 strains were more resistant to both AMPs than ST59 (Figure 4B).

Since biofilm formation is another relevant attribute for nasal colonization, we compared the biofilm formation of the ST7 and ST59. Again, ST7 strains formed more biofilm than ST59 strains (Figure 4C). The increase of ST7 biofilm with the rise of glucose concentration might contribute to the successful colonization of ST7 in diabetic patients with a high FBG level.

### *S. aureus* ST7 is more virulent than ST59

**Figure 5.**
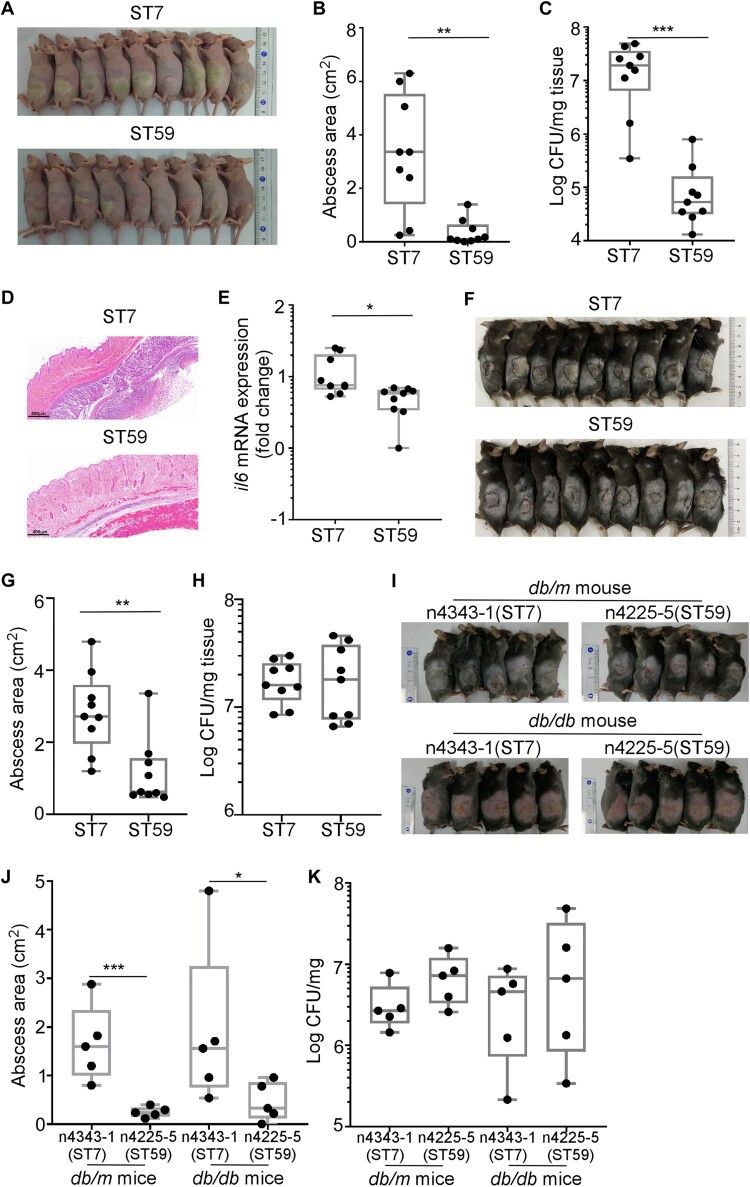
*S. aureus* ST7 cause more severe skin infection compared with ST59 in mouse model. The naked mouse or the non-diabetic mouse (one mouse / isolate, *n* = 9 mice/group) was infected with the test ST7 and ST59 strains (10^8^ CFU) via a subcutaneous injection. Skin abscesses of the infected mice were observed on day 2 after infection. (A) Abscess pictures for naked mouse. (B) Abscess sizes measured 2 d post-infection for naked mouse. (C) Bacterial loads in the infected skin tissues of naked mouse. (D) Histopathological analysis of skin abscess of naked mouse. Representative pictures are shown. (E) Levels of cytokine *il6* gene expression 2 d -abscesses of naked mouse determined by qRT-PCR. (F) Abscess pictures for non-diabetic mouse. (G) Abscess sizes measured 2 d post-infection for non-diabetic mouse. (H) Bacterial loads in the infected skin tissues of non-diabetic mouse. The diabetic mouse model (*n* = 5 mice/group) was infected with a representative strain of ST7 or ST59 (10^8^ CFU) by subcutaneous injection. (I) Abscess pictures. (J) Abscess sizes measured 2 d post-infection. (K) Bacterial loads in the skin tissues. The data were collected from two biological repeats. The statistical significance was measured by unpaired, two-tailed Student’s *t* test. *P < 0.05; **P < 0.01; ***P < 0.001.

Since the colonization of ST7 and ST59 strains seem to be affected by FBG levels, we questioned whether there is an innate difference between the two ST strains. The virulence of ST7 and ST59 isolates were compared by infecting on the skin of hairless mice. Intriguingly, ST7 isolates produced a larger size of skin abscesses, as compared with ST59 isolates at 2 d post-infection (Figure 5A&B). The CFU counting showed that ST7 strains survived better than ST59 strains (Figure 5C). The ST7-infected tissues also showed increased infiltration of inflammantory cells by histopathological analysis (Figure 5D). We also observed the significantly increased IL-6 production in ST7-infected mouse tissues (Figure 5E). The significantly larger size of skin abscesses were also observed in ST7-infected group compared with ST59-infected group at 2 d post-infection using the *db/m* non-diabetic mice (Figure 5F&G). Interestingly, there are no significant differences of bacterial loads between ST7 and ST59-infected groups on the skin of the *db/m* non-diabetic mice (Figure 5H).

The skin infection experiment was also confirmed with the *db/db* diabetic and the *db/m* non-diabetic mice using one randomly selected strain (ST7-n4343-1 and ST59-n4225-5). ST7-n4343-1 also produced larger abscesses than ST59-n4225-5, regardless of the diabetes condition of the mice (Figure 5I&J). Intriguingly, however, the bacterial loads in the infected skin tissues were not significantly different (Figure 5K). Based on these results, we concluded that *S. aureus* ST7 strains are, in general, more virulent than ST59 strains.

### *S. aureus* ST7 have a higher SaeRS activity than ST59

**Figure 6.**
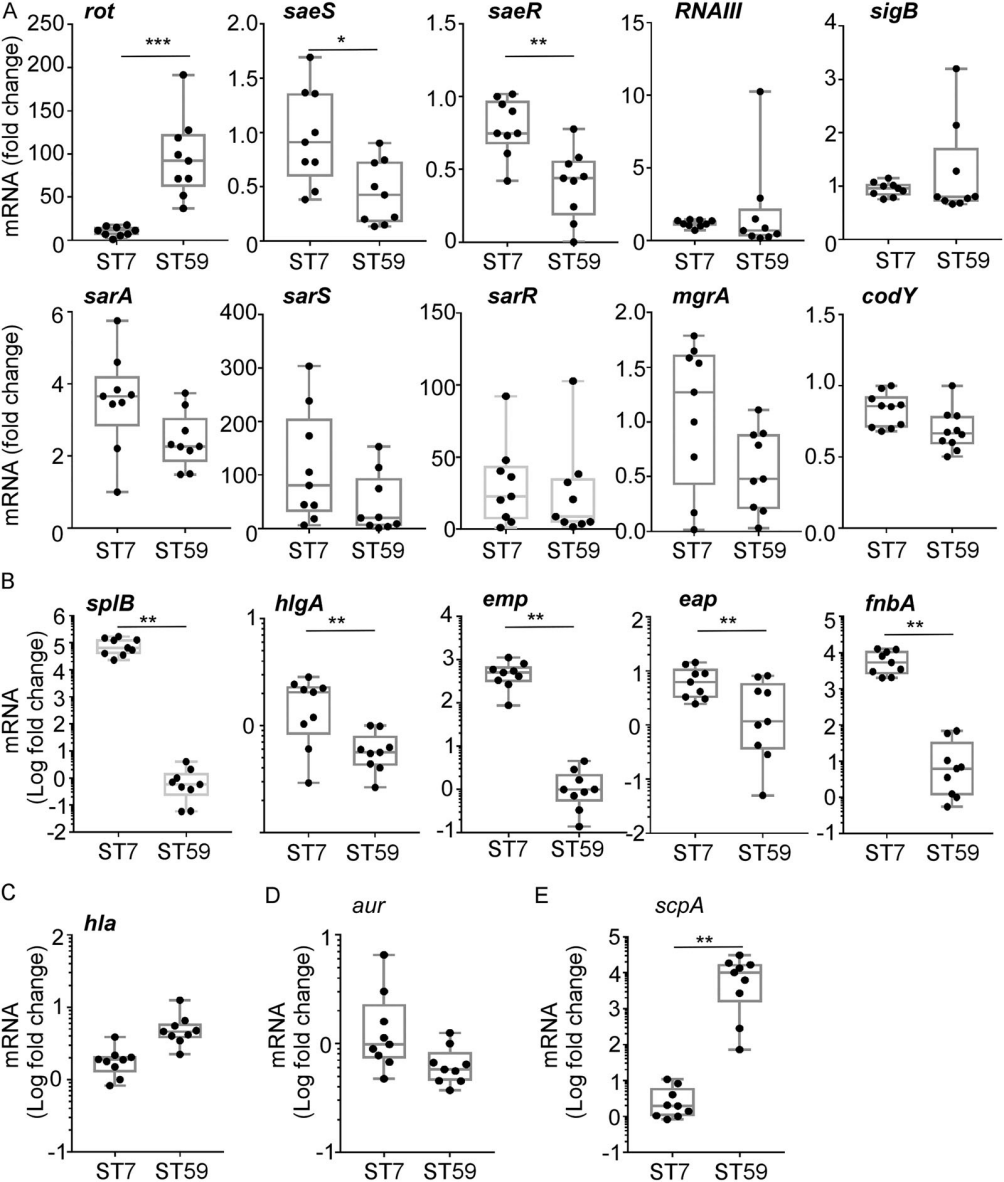
*S. aureus* ST7 strains have a higher SaeRS activity than the ST59 strains. (A) The expression of the main virulence regulators was compared between *S. aureus* ST7 and ST59. (B) The expression of Sae-targets genes with low affinity were compared between *S. aureus* ST7 and ST59. (C) The expression of *hla* with high affinity to SaeRS TCS were compared between *S. aureus* ST7 and ST59. (D/E) The expression of SaeRS-unrelated genes were compared between *S. aureus* ST7 and ST59. The transcript levels were measured by qRT-PCR, using *gyrB* as a reference. The data were collected from three biological repeats. The statistical significance was measured by unpaired, two-tailed Student’s *t* test. *P < 0.05;**P < 0.01; ***P < 0.001.

*S. aureus* ST7 strains appeared to have more virulence traits than ST59 strains (Figure 5). In *S. aureus*, the production of virulence factors is controlled by multiple global regulators [13]. To understand the virulence difference between the ST7 and ST59 strains, we compared the transcript levels of the following 9 global regulators: *rot*, SaeRS two-component system (TCS), *RNAIII*, *sigB*, *sarA*, *sarS*, *sarR*, *mgrA*, and *codY* [27–30]. Of the tested regulators, only *rot* and SaeRS TCS showed a significant difference: the transcription of *rot* was higher in ST59, whereas the transcription of *saeS* and *saeR* was higher in ST7 (Figure 6A). It is well established that *rot* (repressor of toxins) suppresses toxin production, whereas the SaeRS TCS activates it [14,31]. Therefore, it is expected that ST7 is more toxogenic than ST59. To test whether the predominant ST type can represent the strains from different groups, the transcription levels of *rot*, *saeS* and *saeR* were further compared between the strains randomly selected from DM and NDM groups. We observed that *rot* was significantly highly expressed in *S. aureus* strains isolated from NDM group, while the transcription levels of *saeS* and *saeR* were significantly increased in the strains isolated from DM group (Figure S1).

The transcription of the *sae* operon is positively autoregulated [32]. To confirm whether ST7 strains have a higher Sae activity, we compared the transcript levels of five low-affinity (i.e. *splB*, *hlgA*, *emp*, *eap*, and *fnbA*) and a high-affinity (i.e. *hla*) Sae target genes [15], along with a possible target gene (i.e. *aur*) and a non-target gene (i.e. *scpA*). As shown, the transcriptions of all low-affinity target genes were higher in ST7 strains (Figure 6B). On the other hand, the transcription of *hla*, a high-affinity target, was not significantly different (Figure 6C), indicating that the basal Sae activity in ST59 is sufficient to support the full activity of the *hla* promoter. Transcription of *aur*, a possible Sae target, was not significantly different between the two sequence types (Figure 6D). Intriguingly, the non-Sae target gene, *scpA*, showed a higher transcription in ST59 (Figure 6E). Based on these results, we concluded that, overall, ST7 stains have a higher SaeRS activity than ST59 strains.

### The higher SaeRS activity confers a growth advantage to ST7 in high glucose conditions

**Figure 7.**
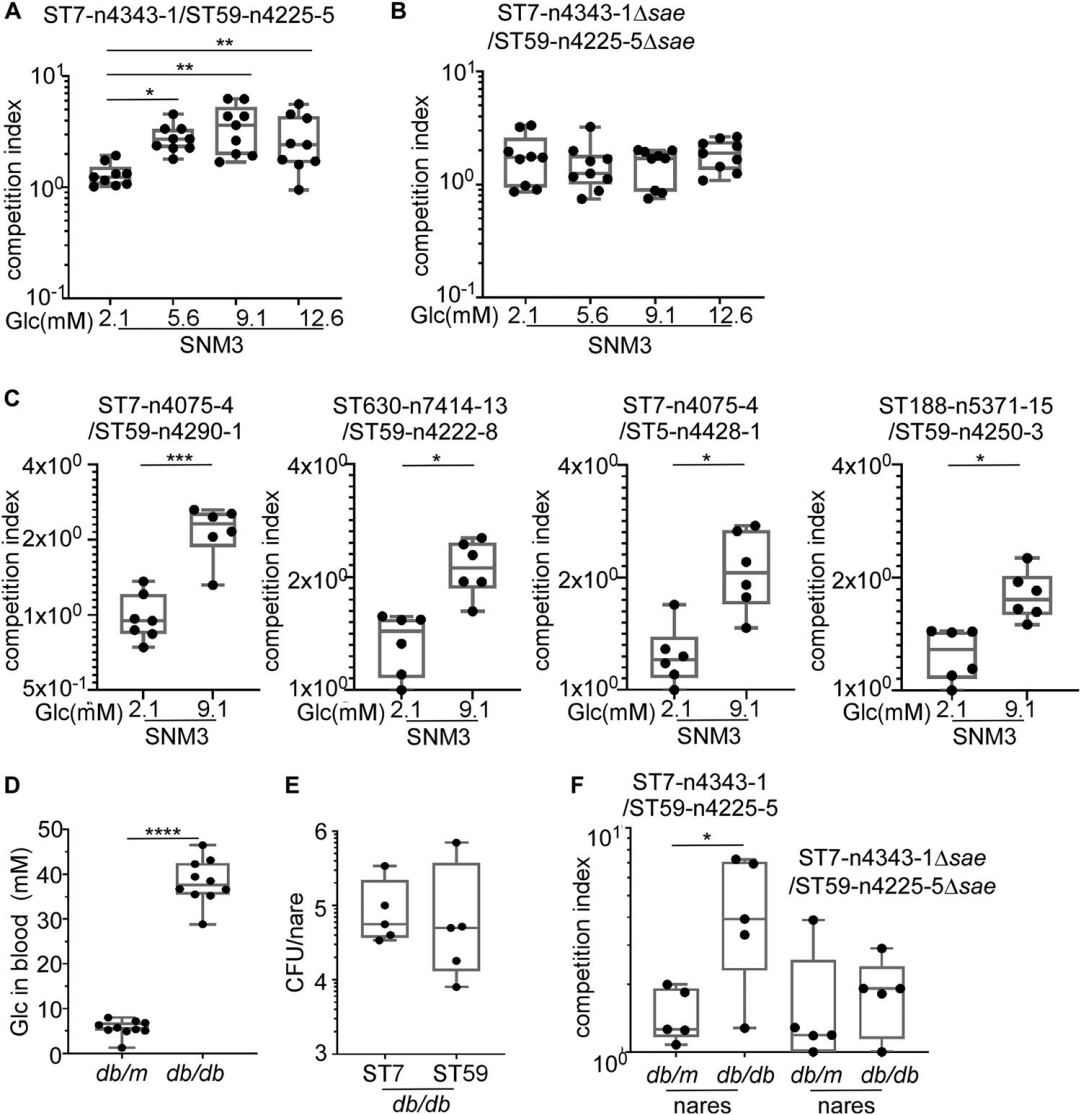
SaeRS is critical for the growth and colonization advantage of *S. aureus* ST7 over ST59 . The competition was tested between ST7-n4343-1 and ST59-n4225-5 (A) and the corresponding *sae* deletion mutant strains (B) by co-culture the two strains in synthetic nasal medium (SNM3). The data were collected from three biological repeats and the statistical significance was measured by one-way ANOVA. (C) The competition was tested between another 4 pairs of strains from DM and NDM group by co-culture each pair of strains in SNM3. The strains were listed in Table S3. The data were polled from two biological repeats with three replicates every time and the statistical significance was measured by unpaired, two-tailed Student’s *t* test. (D) The blood glucose contents were compared between wild-type mouse (*db/m*) and diabetic mouse (*db/db*). (E) The nasal colonization of ST7-n4343-1 and ST59-n4225-5 was compared in diabetic mouse (*db/db*). (F) The competition between ST7-n4343-1 and ST59-n4225-5 and the corresponding *sae* deletion mutant strains was compared between wild-type mouse (*db/m*) and diabetic mouse (*db/db*). The bacterial CFU was determined after co-culture for 6 h in SNM3 or 2 days after nasal colonization *in vivo*. The two strains were differentiated by the resistance to erythromycin. The data for the animal test were repeated twice. The statistical significance was measured by unpaired, two-tailed Student’s *t* test. *P < 0.05; **P < 0.01; ***P < 0.001.

Next, we asked whether the higher Sae activity could provide a growth advantage to ST7 strain over ST59 strains in diabetic conditions. A growth competition assay was carried out using the strain for animal test. To imitate the growth condition, a synthetic nasal medium SNM3, which contains 2.1 mM glucose was used [33]. Without further glucose addition, both strains showed similar growth; however, when the final glucose concentration was adjusted to 5.6, 9.1, or 12.6 mM, ST7-n4343-1 outcompeted ST59-n4225-5 (Figure 7A). When the *sae* operon was deleted, the glucose-dependent growth advantage of ST7-n4343-1 disappeared (Figure 7B). To confirm whether the observed phenotypes from the dominant ST type could reflect the strains from different groups, another four pairs of strains were selected from DM and NDM group with different antibiotic resistance for the competition assay (Table S3). We observed that ST7, ST630 or ST188 from DM group outcompeted ST59 or ST5 from NDM group in higher glucose condition (Figure 7C).

The colonization and growth of the two strains were further compared in diabetic mice. The diabetic (*db*/*db*) mice showed much higher blood glucose concentration than non-diabetic (*db*/*m*) mice (Figure 7D). When the WT strains of ST7-n4343-1 and ST59-n4225-5 were inoculated individually in the nares of diabetic mice, they showed a similar colonization efficiency (Figure 7E). However, when the two strains were mixed and inoculated together, ST7-n4343-1 showed a much higher growth advantage over ST59-n4225-5 in diabetic mice but not in non-diabetic mice (Figure 7F). Intriguingly, when *sae-*deletion mutants were used, the growth advantage of the ST7 strain in diabetic mice was greatly reduced (Figure 7F). To exclude the possibility that the presence of antibiotic resistance affect the results, another pair of ST7-n4075-4 (resistant to erythromycin) and ST59-n4290-1 (sensitive to erythromycin) (Table S3) was used for the competition assay in animal model. We observed that ST7-n4075-4 outcompeted ST59-n4290-1 in diabetic mouse (Figure S2). These results indicate that the higher Sae activity helps ST7 outcompete ST59 in the nasal colonization of diabetic environment.

## Discussion

**Figure 8.**
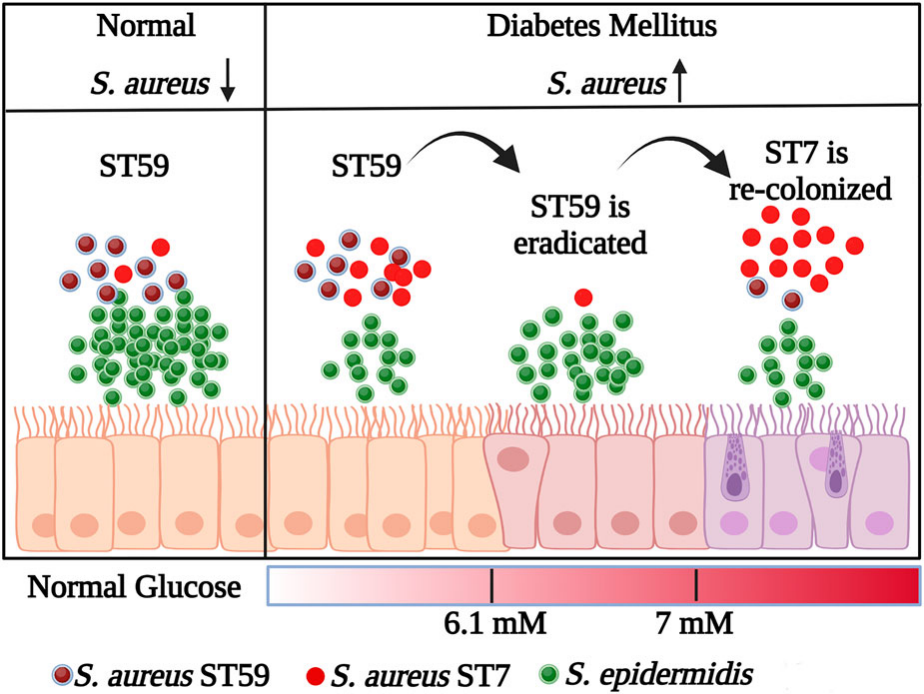
The model for the replacement of *S. aureus* ST types with the increase of glucose. The graphic is created with BioRender.com.

Diabetic foot infections are a significant clinical issue often caused by *S. aureus*, which is commonly found in the nasal passages and a risk factor for staphylococcal infections [34–36]. Our study discovered that diabetic patients have a less diverse nasal microbiome and a higher incidence of *S. aureus* colonization. Additionally, we observed the shift of *S. aureus* ST with the increase of FBG level (Figure 8). The replacement of ST59 by the highly virulent *S. aureus* ST7 poses a potential risk for infection. These findings emphasize the importance of managing blood glucose levels in diabetic patients to decrease the risk of severe *S. aureus* infections.

The microbiome has gained much attention in the prevention and treatment of infectious diseases [37]. In diabetic patients, the diversity of the nasal microbiome is significantly decreased (Figure 1). Although diabetes mellitus causes the reconstruction of the nasal microbiome, the abundance of Staphylococci is only affected by FBG level. In the IGT group of diabetic patients, decreased *S. aureus* colonization occurred with increased colonization of *S. epidermidis* and *Staphylococcus lugdunensis* (Figure 3F). The nasal CoNS play a crucial role in defending against *S. aureus* [38]. *S. lugdunensis* significantly reduced *S. aureus* colonization by producing lugdunin [39]. Nasal colonized *S. epidermidis* produces antimicrobial molecules that defend against *Micrococcus lutues*, *Moraxella catarrhalis*, etc., especially under habitat-specific stress conditions [40]. Unknown antimicrobial peptides produced by *S. epidermidis*, in synergy with LL37, selectively kill *S. aureus* [41]. Moreover, nasal colonized *S. epidermidis* enhances innate immunity by inducing the production of AMPs from human keratinocytes [42]. In our study, only 8 isolates among the tested 318 CoNS displayed antimicrobial activity against *S. aureus* (Figure 4A). This indicates that the inverse correlation between *S. aureus* and CoNS is probably not due to the antimicrobial activity of CoNS.

*S. aureus* ST7 is a prevalent cause of bovine mastitis and can also cause infections in humans [43,44]. It is one of the most common STs found in methicillin-sensitive *S. aureus* and is the second most frequent cause of skin and soft-tissue infections in Shanghai, China [45,46]. ST7 strains have a high prevalence of pore-forming toxins LukED, which are associated with evading the innate immune system [47]. Our study found ST7 to be more virulent than ST59 strains, possibly due to increased activity of the SaeRS TCS (Figures 5 and 6), which activates over 20 virulence factors [44,48]. This higher SaeRS TCS activity may explain the larger skin lesion size, better survival, and higher levels of IL-6 mRNA *in vivo* (Figure 5).

Interestingly, the highly virulent *S. aureus* ST7 also exhibited superior biofilm formation and antimicrobial peptide resistance (Figure 4). Antimicrobial peptide resistance in *S. aureus* is facilitated by modifications of the cell envelope, such as reducing anionic charges by attaching an L-lysine to the major phospholipid phosphatidylglycerol via the multiple peptide resistance factor (MprF) protein, and decreasing negative charges by incorporating D-alanine in teichoic acids through the DltABCD proteins [49–52]. Moreover, the phenol-soluble modulin transporter (Pmt) also confers antimicrobial peptide resistance to *S. aureus* [53]. However, the expression of MprF, DltABCD, and Pmt is not regulated by the SaeRS TCS, which raises the question of the mechanism underlying the superior antimicrobial peptide resistance of ST7. Similarly, the role of the SaeRS TCS in biofilm formation remains unclear. While surface proteins, *ica*-mediated polysaccharides, and extracellular DNA contribute to biofilm formation in *S. aureus*, the SaeRS TCS regulates both biofilm-promoting surface proteins (e.g. Eap) and biofilm-degrading nucleases [54–56]. Furthermore, in the Newman strain, the constitutively active sensor kinase SaeS L18P resulted in poor biofilm formation [57,58]. Therefore, while the SaeRS TCS appears to be crucial for the virulence of ST7, other regulatory systems or factors likely contribute to the colonization advantage of ST7 over ST59.

It appears that glucose enhances the growth and colonization competitiveness of ST7 over ST59 (Figure 7). Glucose is a critical source of energy and carbon for synthesizing cellular components such as amino acids, nucleotides, and lipids, as well as reducing power molecules [59]. *S. aureus* utilizes four glucose transporters to maximize glucose uptake [60]. Additionally, glucose is the primary determinant of regulation by the catabolite control protein (CcpA), which affects not only carbon and amino acid metabolism but also bacterial virulence [61]. It is possible that ST7 strains use glucose more efficiently than ST59 strains, giving them a competitive advantage in higher glucose concentrations. However, the exact molecular mechanism remains to be further elucidated.

One limitation of our study is that the DM group had a significantly higher comorbidity of cerebrovascular diseases and hypertension (Table S3). Cardio-metabolic diseases can impact the human microbiome, and hypertension has been shown to alter the evenness, richness, and diversity of gut microbiome [62]. Therefore, we cannot exclude the possibility that the differences in comorbidity may also contributed to the altered diversity of the nasal microbiome in diabetic patients. Interestingly, among the top 10 genera of the nasal microbiome, *Lactobacillus* was the only genus significantly more abundant in the DM group (Figure 1). *Lactobacillus* is typically considered a probiotic, but inconsistent changes in *Lactobacillus* have been reported in the gut microbiome of diabetic patients, with most studies showing a positive association between *Lactobacillus* and diabetes [63]. As the role of *Lactobacillus* appears to be species-specific, further research at the species level is necessary to clarify the exact role of *Lactobacillus*.

In summary, our results showed that certain ST types of pathogenic bacteria tend to accumulate in the nasal microbiome. The predominant abundance of the highly virulent *S. aureus* ST7 in the nasal microbiome of diabetic patients poses a potential risk for infection. Our results also indicate that the SaeRS TCS regulator is a crucial factor in the competitive fitness of the *S. aureus* ST7 lineage. As such, it is essential to monitor and eliminate nasal *S. aureus* colonization in patients with diabetes and elevated fasting blood glucose levels to ensure proper care.

## Supplementary Material

revised_Supplementary_materials
