# Supplementary material for: Diabetes mellitus promotes the nasal colonization of high virulent Staphylococcus aureus through the regulation of SaeRS two-component system
Source: Emerg Microbes Infect. 2023 Nov 8;12(2):2276335. doi: 10.1080/22221751.2023.2276335 (PMC10796126; doi:10.1080/22221751.2023.2276335)
Supplement: revised_Supplementary_materials [file TEMI_A_2276335_SM2349.docx]

**Supplementary methods**

**SpA typing and Multi-Locus Sequence Typing (MLST).**

The *spA* polymorphic X region and seven housekeeping genes: carbamate kinase (*arcC*), shikimate dehydrogenase (*aroE*), glycerolkinase (*glp*), guanylate kinase (*gmk*), phosphate acetyltransferase (*pta*), triosephosphate isomerase (*tpi*), and acetyl coenzyme A acetyltransferase (*yqiL*), were amplified by PCR. The sequences of the PCR products were used for *spA* typing (<http://spa.ridom.de/>), or were compared with the existing sequences available in the MLST website database (<http://www.pubmlst.net>). The allelic numbers for PCR sequences were determined by the existing sequences available at the MLST website (http://www.mlst.net). All the primers werer listed in **Table S2**.

**Antimicrobial activity of CoNS.**

Totally, 240 *S. epidermidis* and all the 78 other coagulase-negative Staphylococi, were used for the agar diffusion (Kirby-Bauer) antimicrobial activity assay. All bacteria were cultured in TSB at 37 °C with shaking at 180 rpm for 12 h. The bacteria were stamped on tryptic soy agar (TSA) plates inoculated with the relevant test strains (*S. aureus*) and incubated at 37 °C for 24 h. To prepare *S. aureus* test plates, autoclaved TSA was cooled to 45 °C and inoculated with the test strains to an OD_600_ = 0.001 for *S. aureus*. After mixing thoroughly, plates of 50 ml volume were poured.

**Killing assay *in vitro*.**

The overnight culture was diluted into fresh TSB (1:100) and grown to OD_600_ = 2.0. After centrifugation, the bacteria pellets were washed twice with 10 mM sodium phosphate buffer (pH 6.5) including 100 mM NaCl, and resuspended in the same buffer with or without AMPs to a final concentration of 10^6^. The concentrations of 75 μg/ml (hBD3 or LL37, GenScript, USA) were incubated with bacteria at 37 °C for 3 h. Then, the survival bacteria were plated on TSB agar plates by series dulution.

**Semiquantitative biofilm formation assay.**

The overnight cultures of *S. aureus* isolates were diluted 1:100 into fresh TSB with different concentration of glucose. The diluted cultures were then pipetted into sterile 96-well flat-bottom tissue culture plates. After incubation at 37 °C for 24 h, the cells were fixed by Bouin’s fixative for at least 1 h. The wells were washed with PBS after removal of fixative, stained with crystal violet, and washed with slow-running water. After drying, biofilm formation was measured with a MicroELISA autoreader (BioTek, Synergy 2) at 570 nm.

**The determination of blood glucose concentration.**

The mouse blood serum was collected by centrifugation at 1500 g for 10 min. The blood glucose contents were measured with Pureauto S GLU (Hexokinase UV method) by using Cobas e801 (Roche Diagnostics Co., Ltd), an automated analyzer for blood tests.

**Mouse nasal colonization model.**

Overnight bacterial cultures were diluted 1:100 in fresh TSB and grown for 4 h at 37 °C. Bacteria were harvested by centrifugation at 4000 × g for 10 min, washed twice, and suspended in PBS. *db*/*db* mice (n=5 mice/group) were anesthetized by intraperitoneal injection with Avertin (Sigma T48402), and 20 μl aliquots containing 1×10^7^ CFU were instilled equally split between the two nostrils. The animals were sacrificed at the 2^nd^ day and the noses were surgically removed. The noses were homogenized in 0.5 ml of PBS on ice using a manual homogenizer (Tiagen), the homogenized tissues were diluted and then plated on 5% sheep blood agar for CFU determination.


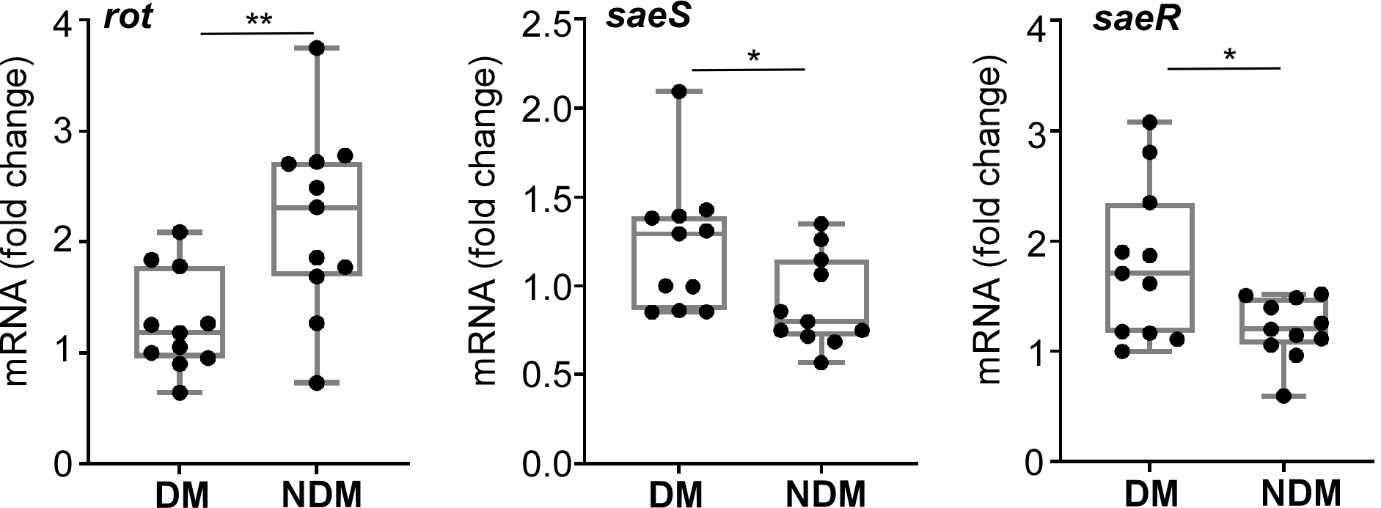


**Figure S1**. ***S. aureus* strains from DM group have a higher SaeRS activity than those form NDM group.** The expression of *rot*, *saeS* and *saeR* was compared between *S. aureus* ST7 and ST59.


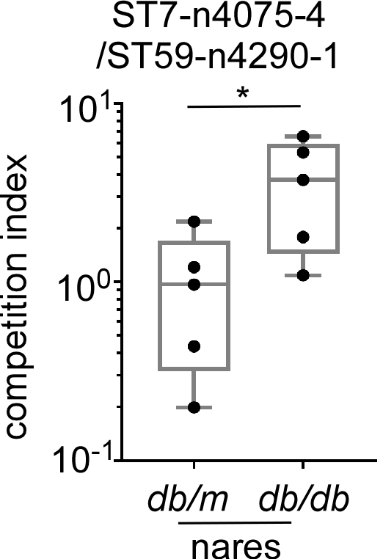


**Figure S2**. **ST7-n4075-4 outcompeted ST59-n4290-1 *in vivo*.** The competition between ST7-n4075-4 and ST59-n4290-1 strains was compared between wild-type mouse (*db/m*) and diabetic mouse (*db/db*). The bacterial CFU was determined after co-culture for 6 h in SNM3 or 2 days after nasal colonization *in vivo*. The two strains were differentiated by the resistance to erythromycin. The data for the animal test were repeated twice. The statistical significance was measured by unpaired, two-tailed Student’s t test. *, *P* < 0.05.

| **Table S1. The selected 120 *S. aureus* isolates for MLST.** | | | |
| --- | --- | --- | --- |
| **DM group** | | | |
| **NO** | **DM** | **spA typing** | **MLST** |
| 1 | n84X-8 | t189 | ST188 |
| 2 | n84X-9 | t189 | ST188 |
| 3 | n84X-13 | t189 | ST188 |
| 4 | n84X-18 | t189 | ST188 |
| 25 | n4012-1 | NO spa type | ST1 |
| 36 | n4012-14 | t127 | ST1 |
| 42 | n4075-4 | t091 | ST7 |
| 51 | n4238-17 | t437 | ST59 |
| 55 | n4248-3 | t091 | ST7 |
| 60 | n4248-8 | NO spa type | ST7 |
| 94 | n4343-1 | t091 | ST7 |
| 95 | n4343-7 | t7568 | ST7 |
| 97 | n4343-13 | t127 | ST59 |
| 108 | n5018-11 | t605 | ST7 |
| 109 | n5018-14 | t605 | ST7 |
| 110 | n5018-17 | t605 | ST7 |
| 111 | n5018-18 | t605 | ST7 |
| 132 | n5297-7 | NO spa type | ST7120 |
| 133 | n5351-4 | NO spa type | ST398 |
| 134 | n5351-5 | NO spa type | ST398 |
| 135 | n5351-11 | t011 | ST398 |
| 136 | n5351-12 | t011 | ST398 |
| 137 | n7392-20 | t4549 | ST630 |
| 138 | n7414-2 | t4549 | ST630 |
| 143 | n7414-13 | t4549 | ST630 |
| 145 | n8503-3 | t172 | ST59 |
| 146 | n8503-6 | t172 | ST59 |
| 147 | n8503-10 | t172 | ST59 |
| 148 | n8503-13 | t172 | ST59 |
| 150 | n8503-20 | t172 | ST59 |
| 152 | n8505-10 | t172 | ST59 |
| 156 | n8505-20 | t172 | ST59 |
| 157 | n8707-18 | t172 | ST59 |
| 158 | n9536-1 | t091 | ST7 |
| 159 | n9536-3 | t091 | ST7 |
| 161 | n9536-15 | t091 | ST7 |
| 164 | n9558-14 | t5554 | ST1821 |
| 165 | n9577-1 | t605 | ST7 |
| 166 | n9577-2 | t034 | ST398 |
| 168 | n9577-4 | t034 | ST398 |
| 169 | n9577-6 | t605 | ST7 |
| 170 | n9577-7 | t605 | ST7 |
| 171 | n9577-8 | t034 | ST398 |
| 172 | n9577-9 | t034 | ST398 |
| 174 | n9577-11 | t034 | ST398 |
| 175 | n9577-13 | t605 | ST7 |
| 176 | n9577-14 | t605 | ST7 |
| 177 | n9577-16 | t605 | ST7 |
| 178 | n9577-17 | t034 | ST398 |
| 179 | n9577-18 | t034 | ST398 |
| 180 | n9577-19 | t605 | ST7 |
| 181 | n9577-20 | t605 | ST7 |
| 182 | n5371-3 | t189 | ST188 |
| 183 | n5371-4 | t189 | ST188 |
| 184 | n5371-6 | t189 | ST188 |
| 185 | n5371-11 | t189 | ST188 |
| 186 | n5371-12 | t189 | ST188 |
| 187 | n5371-15 | t189 | ST188 |
| 188 | n5371-20 | t189 | ST188 |
| 189 | n4264-8 | t002 | ST2590 |
| **NDM group** | | | |
| **NO** | **DM** | **spA typing** | **MLST** |
| 5 | n4007-1 | t377 | ST360 |
| 11 | n4007-7 | t377 | ST360 |
| 43 | n4213-19 | t437 | ST59 |
| 44 | n4218-15 | t437 | ST59 |
| 45 | n4219-8 | t437 | ST59 |
| 46 | n4222-8 | t437 | ST59 |
| 48 | n4224-7 | t437 | ST59 |
| 49 | n4225-5 | t437 | ST59 |
| 50 | n4231-1 | t034 | ST398 |
| 52 | n4247-16 | t437 | ST59 |
| 53 | n4247-20 | t437 | ST59 |
| 73 | n4250-3 | t437 | ST59 |
| 74 | n4254-16 | t437 | ST59 |
| 75 | n4290-1 | t172 | ST59 |
| 76 | n4290-2 | t172 | ST59 |
| 77 | n4290-3 | t172 | ST59 |
| 78 | n4290-4 | t172 | ST59 |
| 79 | n4290-5 | t172 | ST59 |
| 80 | n4290-7 | t172 | ST59 |
| 81 | n4290-8 | t172 | ST59 |
| 82 | n4290-9 | t172 | ST59 |
| 83 | n4290-10 | t172 | ST59 |
| 84 | n4290-11 | t172 | ST59 |
| 85 | n4290-12 | t172 | ST59 |
| 86 | n4290-13 | t172 | ST59 |
| 87 | n4290-14 | t172 | ST59 |
| 88 | n4290-15 | t172 | ST59 |
| 89 | n4290-16 | t172 | ST59 |
| 90 | n4290-17 | t172 | ST59 |
| 91 | n4290-18 | t172 | ST59 |
| 92 | n4290-19 | t172 | ST59 |
| 93 | n4290-20 | t172 | ST59 |
| 98 | n4398-1 | NO spa type | ST7 |
| 99 | n4428-1 | t548 | ST5 |
| 100 | n4428-2 | t548 | ST5 |
| 101 | n4428-3 | t548 | ST5 |
| 102 | n4428-4 | t548 | ST5 |
| 103 | n4428-5 | t548 | ST5 |
| 104 | n4428-6 | t548 | ST5 |
| 105 | n4428-7 | t548 | ST5 |
| 106 | n4428-8 | t548 | ST5 |
| 107 | n4428-9 | t548 | ST5 |
| 112 | n5047-1 | t3932 | ST7 |
| 113 | n5047-3 | t3932 | ST7 |
| 114 | n5047-4 | t3932 | ST7 |
| 115 | n5058-3 | t011 | ST398 |
| 116 | n5058-6 | t011 | ST398 |
| 118 | n5058-9 | t011 | ST398 |
| 119 | n5058-10 | t011 | ST398 |
| 120 | n5058-12 | t011 | ST398 |
| 121 | n5058-14 | t011 | ST398 |
| 123 | n5058-16 | t011 | ST398 |
| 124 | n5058-17 | t011 | ST398 |
| 125 | n5058-18 | t011 | ST398 |
| 126 | n5058-20 | t011 | ST398 |
| 127 | n5073-1 | t091 | ST7 |
| 128 | n5073-12 | t091 | ST7 |
| 129 | n5073-15 | t091 | ST7 |
| 130 | n5073-18 | t091 | ST7 |
| 131 | n5073-20 | t091 | ST7 |

**Table S2. Oligonucleotides used in this study.**

| **Name** | **Sequence (5’ 🡪 3’)** | **Target** |
| --- | --- | --- |
| *nuc*-F | TGTAGTTTCAAGTCTAAGTAGCTCAGCAA | For taqman probe |
| *nuc*-R | TGCACTATATACTGTTGGATCTTCAGAA | For taqman probe |
| *spA*-F | TAAAGACGA TCCTTCGGTGAGC | For *spA* typing |
| *spA*-R | CAGCAGTAGTGCCGTTTGCTT | For *spA* typing |
| *arc*-F | TTG ATT CAC CAG CGC GTA TTG TC | For MLST |
| *arc*-R | AGG TAT CTG CTT CAA TCA GCG | For MLST |
| *aroE*-F | ATC GGA AAT CCT ATT TCA CAT TC | For MLST |
| *aroE*-R | GGT GTT GTA TTA ATA ACG ATA TC | For MLST |
| *glpF*-F | CTA GGA ACT GCA ATC TTA ATC C | For MLST |
| *glpF*-R | TGG TAA AAT CGC ATG TCC AAT TC | For MLST |
| *gmk*-F | ATC GTT TTA TCG GGA CCA TC | For MLST |
| *gmk*-R | TCA TTA ACT ACA ACG TAA TCG TA | For MLST |
| *pta*-F | GTT AAA ATC GTA TTA CCT GAA GG | For MLST |
| *pta*-R | GAC CCT TTT GTT GAA AAG CTT AA | For MLST |
| *tpi*-F | TCG TTC ATT CTG AAC GTC GTG AA | For MLST |
| *tpi*-R | TTT GCA CCT TCT AAC AAT TGT AC | For MLST |
| *yqiL*-F | CAG CAT ACA GGA CAC CTA TTG GC | For MLST |
| *yqiL*-R | CGT TGA GGA ATC GAT ACT GGA AC | For MLST |
| *rot*-F | GGTTCTATGACGCTTAAAGAAATG | For RT-PCR |
| *rot*-R | CGTTCTCGTACGCTTATACGG | For RT-PCR |
| *saeR*-F | ATG ACC CAC TTA CTG ATC GT | For RT-PCR |
| *saeR*-R | GAA ATT GCT TCT TTA CCG CT | For RT-PCR |
| *saeS*-F | GATGATATGACTCATATTATCAC | For RT-PCR |
| *saeS*-R | CGTTACAGAAATTCACTTCTAATG | For RT-PCR |
| *RNAIII*-F | ATAGCACTGAGTCCAAGGAAACTAACT | For RT-PCR |
| *RNAIII*-R | GCCATCCCAACTTAATAACCATGT | For RT-PCR |
| *sigB*-F | GGAGTGTACATGTTCCGAGACG | For RT-PCR |
| *sigB*-R | CCATTGCTTCTAACACTTCTTC | For RT-PCR |
| *sarA*-F | GCTGTATTGACATACATCAGCG | For RT-PCR |
| *sarA*-R | CATGCTCATTACGTTTTTTATCG | For RT-PCR |
| *sarS*-F | CAACGAGAGAAAATTGCAGAAC | For RT-PCR |
| *sarS*-R | GGTATCATCTGTGATTCACTTTG | For RT-PCR |
| *sarR*-F | AACTGTTCTTTCGTCTTGTAAACT | For RT-PCR |
| *sarR*-R | TGCTCAGAGTTCAAACCTTACT | For RT-PCR |
| *mgrA*-F | GCTCAAAGACAAGTTAATCGCTAC | For RT-PCR |
| *mgrA*-R | CGTTTACAGGAGATTCATCCCA | For RT-PCR |
| *codY*-F | CTAGGTGAATATGCTGCTACAG | For RT-PCR |
| *codY*-R | CCATTGTAATAGCAGCTTTATCG | For RT-PCR |
| *splB*-F | GCGTGCAATAGAACGTGGACC | For RT-PCR |
| *splB*-R | CAATACTGCTACCTTCTACTG | For RT-PCR |
| *hlgA*-F | GGTGCAGAAATCATCAAAAGAAC | For RT-PCR |
| *hlgA*-R | GTCTGAATATGTTGTTCTAGAG | For RT-PCR |
| *emp*-F | CACGCTAAAGCATCAGTGACAG | For RT-PCR |
| *emp* -R | CTAGCTGCCGATGAATCTGC | For RT-PCR |
| *eap*-F | CAGTACTGAAGCATGATAGAG | For RT-PCR |
| *eap* -R | CTCTTAACATCTTTCGCATGAAC | For RT-PCR |
| *fnbA* F | TCGCGTTACTAGCTTCCTTAGCTT | For RT-PCR |
| *fnbA* R | CAGCCAAGAACGGCATCAG | For RT-PCR |
| *hla*-F | AATAACTGTAGCGAAGTCTGGTGAAA | For RT-PCR |
| *hla*-R | GCAGCAGATAACTTCCTTGATCCT | For RT-PCR |
| *aur*-F | GCCTAGAAGATTTAACGCATC | For RT-PCR |
| *aur*-R | CATTTTGTCACCGATCCATGCG | For RT-PCR |
| *scpA*-F | GGTTACTCAAAATCATAAAGC | For RT-PCR |
| *scpA* -R | GTTTCTCTTATCTTGAAATTCTC | For RT-PCR |
| *gyrB*-F | CAAATGATCACAGCATTTGGTACAG | For RT-PCR |
| *gyrB*-R | CGGCATCAGTCATAATGACGAT | For RT-PCR |

**Table S3. The strains selected for competition assay *in vitro* used in this study.**

| Groups | DM | NDM |
| --- | --- | --- |
| A | ST7-n4343-1 (Erm^S^) | ST59-n4225-5 (Erm^R^) |
| B | ST7-n4075-4 (Erm^R^) | ST59-n4290-1 (Erm^S^) |
| C | ST630-n7414-13 (Erm^S^) | ST59-n4222-8 (Erm^R^) |
| D | ST7-n4075-4 (Erm^R^) | ST5-n4428-1 (Erm^S^) |
| E | ST188-n5371-15 (Erm^S^) | ST59-n4250-3 (Erm^R^) |

Erm^S^: Sensitive to erythromycin (10 μg/ml); Erm^R^: Resistant to erythromycin (10 μg/ml)

**Table S4. Comorbidity in the NDM and the DM groups.**

| Disease | **NDM** | **DM** | ***P* value** |
| --- | --- | --- | --- |
| chronic pulmonary disease | 16.13% | 16.67% |  |
| chronic heart disease | 11.29% | 16.67% |  |
| chronic renal disease | 2.15% | 4.55% |  |
| chronic liver failure | 60.75% | 70.20% |  |
| cerebrovascular disease | 8.60% | 20.20% | **0.002 |
| neoplastic disease | 3.23% | 5.56% |  |
| hypertension | 48.39% | 62.63% | **0.007 |
| neurological disease | 0.0% | 0.51% |  |
| digestive system disease | 22.58% | 27.78% |  |
